# Nebivolol ameliorates sepsis-evoked kidney dysfunction by targeting oxidative stress and TGF-β/Smad/p53 pathway

**DOI:** 10.1038/s41598-024-64577-5

**Published:** 2024-06-26

**Authors:** Rahma Tharwat Sabra, Amany Abdlrehim Bekhit, Nourhan Tharwat Sabra, Nadia Ahmed Abd El-Moeze, Moustafa Fathy

**Affiliations:** 1 Department of Biochemistry, Faculty of Pharmacy, Minia University, Minia, 61519 Egypt; 2 Department of Anatomy and Embryology, Faculty of Medicine, Beni-Suef University, Beni-Suef, 62514 Egypt; 3 Department of Pathology, Faculty of Medicine, Beni-Suef University, Beni-Suef, 62514 Egypt

**Keywords:** Nebivolol, Sepsis, Nephrotoxicity, Fibrosis, TGF-β/Smad/p53, NF-kB, Drug discovery, Gastroenterology, Nephrology

## Abstract

Sepsis is a potential fetal organ destruction brought on through an overzealous immunologic reaction to infection, causing severe inflammation, septic shock, and damage to different organs. Although there has been progress in the identification and controlling of clinical sepsis, the fatality rates are still significant. This study, for the first time, intended to examine the possible ameliorative impact of Nebivolol, a β1-adrenergic antagonist antihypertensive drug, against nephrotoxicity resulted from cecal ligation and puncture (CLP)-induced sepsis in rats, on molecular basis. Sixty male Wistar albino rats were chosen. Oxidative stress indicators and biochemical markers of kidney activity were evaluated. Inflammatory mediators, fibrosis- and apoptosis-related proteins and gene expressions were investigated. Moreover, renal histopathological investigation was performed. CLP-induced nephrotoxicity characterized by markedly elevated serum levels of creatinine, blood urea nitrogen, uric acid, and renal malondialdhyde. On the other hand, it decreased serum total protein level, renal superoxide dismutase activity and reduced glutathione level. Additionally, it significantly elevated the renal inflammatory mediators (tumor necrosis factor-alpha, ilnerlukin (IL)-6, and IL-1β) and Caspase-3 protein, reduced IL-10 level, amplified the expression of transforming growth factor-beta 1 (TGF-β1), p-Smad2/3 and alpha-smooth-muscle actin proteins, downregulated the *B cell lymphoma-2 (Bcl-2)* gene and elevated the transcription of *Bcl-2-associated X-protein (Bax)*, *p53* and *Nuclear factor-kappa B* (*NF-κB*) genes. Furtheremor, kidney tissues exhibited significant histopathological changes with CLP. On the contrary, Nebivolol significantly improved all these biochemical changes and enhanced the histopathological alterations obtained by CLP. This research showed, for the first time, that Nebivolol effectively mitigated the CLP-induced kidney dysfunction via its antioxidant, antifibrotic and anti-apoptotic activity through modulation of oxidative stress, TGF-β/NF-κB and TGF-β/Smad/p53 signaling pathways.

## Introduction

Sepsis is a frequent consequence of trauma and injuries, which remains a burdensome public health problem globally. Aggressive immune defense against infection, severe inflammation, sharp drop in blood pressure, and tissue and organ damage are the hallmarks of sepsis, a potentially fatal organ failure^1,2^. With the support of the Surviving Sepsis Campaign (SSC), the mortality rate from sepsis has decreased; nevertheless, the total number of deaths from sepsis has increased due to the increasing number of new cases^3^. Around 2017, 48.9 million instances were reported, with approximately 1.7 million new cases annually and nearly 11 million deaths, which is almost 20% of total global deaths, with the highest load on nations with moderate and low incomes, especially Africa^4^. The known methods for handling sepsis, including antibiotics use, organ support therapy, or fluid resuscitation, have poor predicting influence on patient health with sepsis. Nowadays, sepsis is deemed to be the leading cause of death, surpassing even cancer or coronary diseases, and raises the expense of medical care in the intensive care units^5^.

Acute kidney injury (AKI) is an established organ malfunction induced by sepsis; it affects 40–50% of cases^6,7^. Sepsis-associated AKI (S-AKI) is associated with a six-to eight-fold greater probability of death in sepsis patients and a higher prevalence of chronic kidney dysfunction (CKD) progression in sepsis survivors^8^. Until now, the mechanisms of action responsible for initiating and developing kidney damage have not been fully understood. So, it is a priority to discover novel strategies and their fundamental mechanisms for enhancing the consequences of sepsis patients with renal damage.

Renal fibrosis, characterized by a buildup of fibroblasts, excess matrix proteins, and the failure of functioning nephrons, is a significant pathological feature of progressive kidney damage^9^. Numerous studies have demonstrated that various markers, including cytokines, growth factors, and stress species molecules, mediate advancing renal fibrosis by intercalating between various pathways. It has been established that transforming growth factor-β1 (TGF-β1) is a fundamental mediator in apoptosis, inflammation, and renal fibrosis pathogenesis^9–11^.

TGF-β1 was found to exert its pathological effect via the smooth muscle actin (SMA) and non-canonical signaling cascades, which control the transcription of target genes, including the inflammatory, fibrotic, and apoptosis mediators, such as NF-κB, Smad, and p53, and their implication in epithelial-mesenchymal transition (EMT) stimulation^12,13^. All these observations make the crosstalk between TGF-b1/Smad/p53/NF-kB to be critical inflammatory, apoptotic and fibrogenic cascade that modulates the progression of renal fibrosis^14,15^.

Repurposing drugs^16–18^ and looking for new pharmacological properities for natural^19–21^ or synthetic^22–24^ agents to discover novel strategies for treating various disorders became necessary^25,26^. Nebivolol is a selective third-generation β1-adrenoreceptor antagonist. It enhances renal blood circulation and the rate of glomerular filtration (GFR) as it produces nitric oxide (NO), which has a vasodilator effect by activating b3-adrenergic receptor. Furthermore, it exhibits potent anti-oxidant activity by direct scavenging activity on reactive oxygen species via inhibition of the NADPH oxidase system^27,28^. Nebivolol reduces oxidative stress by blocking nuclear factor-κB (NF-kB) activation, which in turn lessens the inflammatory mediators^29^. Furthermore, Nebivolol's NO-regulating action demonstrates an impressive anti-apoptotic characteristic^30^.

As far as we know, no prior research has been carried out to examine the possible Nebivolol's renal fibrosis-fighting properties against cecal-ligation and puncture (CLP)-induced sepsis. This study, for the first time, aimed to examine the possible ameliorative impact of Nebivolol against kidney dysfunction resulted from CLP-induced sepsis in rats, and investigate the molecular mechanism underlying this effect.

## Material and methods

### Drugs and chemicals

Nebivolol hydrochloride was donated from Marcyrl Pharmaceutical Industries (Cairo, Egypt) and was suspended in 1% to tween 80 in distilled water. Vitamin C was acquired from Epico Pharmaceutical company (Cairo, Egypt) and dispersed in normal saline. All drugs were dissolved directly before use. 10× PBS (pH 7.4) (#AM9624, Invitrogen, Life Technologies Ltd, UK) was used. Unless otherwise noted, all commercially accessible chemicals had the highest analytical performance.

### Experimental model

As previously secribed, the CLP model was used to provoke sepsis^31^. Briefly, a combination of xylazine (10 mg/kg bw) and ketamine (80 mg/kg bw) was injected intraperitoneally to sedate the rats. Rats' abdomen walls were cleansed and shaved using a 10% povidone-iodine solution^32^. An incision took place in the bottom left region of the abdomen. The cecum was exteriorized and tied regularly with 0.3 mm silk medical thread at 75% of its length. The ligated part of the cecum was twice punctured through and through using an 18-gauge injection needle. The identical interventions without CLP were performed to the sham rats.

### Animals and the experimental schedule

Seven-week-old, sixty healthy Wistar male albino rats weighting 180–200 g were obtained from the National Research Center in Giza, Egypt and kept in an experimental animal laboratory with temperature control, free from the pathogen with an unrestricted supply of regular laboratory food and water. The experiment was accepted and executed in compliance with the Care and Use of Laboratory Animals standard operating procedures approved by the Research Ethics Committee, Minia University (Project code: MPEC (2301202)).

Following a week of housing, rats were randomly placed into six groups of ten each.

Group I (Sham group): rats were given two intraperitoneal injections: one of normal saline (*i.p.*) and the other of 1% tween 80 in distilled water (*i.p.*). After one hour, the same CLP surgical procedure was performed without CLP induction.

Group II (Nebivolol 10): rats obtained Nebivolol (10 mg/kg) *i.p.*, as a single dose.

Group III (CLP): Rats obtained a single intraperitoneal injection of normal saline. After one hour, a CLP operation was performed.

Group IV (CLP/Nebivolol 4): rats obtained a single dose of Nebivolol (4 mg/kg) *i.p*. one hour before the CLP operation^33–35^.

Group V (CLP/Nebivolol 10): rats obtained Nebivolol (10 mg/kg) *i.p.* as a single dose one hour before the CLP operation^36,37^.

Group VI (CLP/Vitamin C): an hour following the CLP procedure, rats obtained a single intraperitoneal (*i.p.)* dosage of Vitamin C^38^. This group was used as a positive control group.

Rats were given isoflurane anesthesia twenty-four hours after the CLP surgery. Blood was extracted from the jugular vein by slaughtering the rats, and serum was collected to analyze the biological markers. After being removed, the kidneys were cleaned in cold saline and divided into four sections. For histological investigation, one part was immersed in 10% formaldehyde. The second part was homogenized in 1X PBS, pH 7.4, to quantify the kidney's level of oxidative stress markers. The third piece was used for ELISA and western blotting studies, while the remaining part was used for quantitative RT-PCR.

### Biochemical measurements

Serum total protein, blood urea nitrogen (BUN), and Creatinine (Cr) were calculated using commercial kits (#230601, #230603, and #230104) from Bio Diagnostics company (Giza, Egypt). Additionally, serum uric acid was measured with the kit from BioMed Pharmaceutical Industries (Cairo, Egypt) (#UA 121120). Every measurement was taken firmly following the kit's instructions.

### Invertigation of kidney oxidative stress

Samples of kidney tissue were homogenized in 1X PBS. The tissue weight to PBS ratio was one to five. The homogenates underwent a 10-min, 16,000×*g* centrifugation at 4 °C. Superoxide dismutase (SOD) activity, reduced glutathione (GSH) level, and malondialdehyde (MDA) content were determined,in the supernatant, in accordance with manufacturer instructions utilizing the kits acquired from Bio Diagnostics company in Giza, Egypt (#SD 1019, #GR 0604, #MD 1304, respectively).

### Inflammation and apoptosis parameters in kidney tissues

Rat TNF-α, IL-1β , IL-6 and IL-10 kidney tissue cytokines were assessed using the following kits: rat *TNF-α* ELISA kit (#438206, BioLegend, San Diago, CA, USA), rat *IL-1β* ELISA kit (#E0119Ra, Bioassay Technology Laboratory, Birmingham, UK), rat *IL-6* ELISA kit (#SEA079Ra, Cloud-Clone Crop, Houston, TX, USA) and rat *IL-10* ELISA kit (#SEA056Ra, Cloud-Clone Crop, Houston, TX, USA). Also, a rat Caspase-3 ELISA kit (#E459-100, Bio Vision Inc., Milpitas, CA, USA) was used to quantify Caspase-3 protein in the kidney homogenates, following the guidelines provided by the manufacturer. Using an ELISA plate reader (Stat Fax 2200, Awareness Technologies, Florida, USA), the OD range used to measure color absorbance was 490–630 nm. The protein level within the tissue was estimated using a Bradford protein colorimetric kit (#E-BC-K168-S, Elabscience Inc., USA), and the results were expressed as pg/mg tissue protein.

### Quantitative real-time polymerase chain reaction

In accordance with the manufacturer's guidelines, kidney tissues were treated for the total RNA extraction using a direct-zol™ RNA Miniprep Plus kit (#R2072, Zymo research crop, USA), and the quality of the extracted RNA was evaluated through the use of a Thermo Fisher Scientific, USA, Nanodrop 8000 spectrophotometer. The SuperScript IV One-Step RT-PCR kit (#12594100, Thermo Fisher Scientific, Waltham, MA, USA) was employed to perform quantitative polymerase chain reactions (qPCR) using equivalent quantities of total RNA in duplicate. The kit allows c-DNA synthesis and PCR amplification to be performed in a single reaction tube, as the supplier recommends, on a Step One Real-Time PCR system (Applied Biosystems, Foster, USA). Melt curve studies proved the selectivity of the amplification. The endogenous reference gene *β-actin,* was used to standardize the target genes' cycle threshold (Ct) values. The 2^−∆∆Ct^ method was then applied to divide the expression in each sample by the sham sample to determine the fold change.

The precise primer sequences used with their accession numbers for *Bax, Bcl-2, p53, NF-*κ*B and β-actin* genes are displayed in Table 1.

**Table 1:** Primer sequences and their accession numbers used in qRT-PCR.

Genes		Primer sequence (5′–3′)	Accession number
*Bax*	Forward	CCGGCAGGCCCATACTGAAT	AB046392.1
	Reverse	CTTGGACAGGGCAGATAGCC	
*Bcl-2*	Forward	TGATAACCGGGAGATCGTGA	S74122.1
	Reverse	TCGCCAACGCTGGGCCTGCG	
*p53*	Forward	TGGGTCACCTCCACACCTCC	XM_032912337.1
	Reverse	GGATGTTGCAGAGTTGTTAG	
*NF-κB*	Forward	GTCTCAAACCAAACAGCCTCAC	NM_199267.2
	Reverse	CAGTGTCTTCCTCGACATGGAT	
*β-actin*	Forward	TGTCACCAACTGGGACGATA	XM_039089807.1
	Reverse	ACCCTCATAGATGGGCACAG	

### Western blotting analysis

Kidney homogenate samples with equal protein portions (20 µg) were isolated using 12.5% sodium dodecyl sulfate–polyacrylamide gel electrophoresis (SDS-PAGE) and placed onto a PVDF membrane. After treating the membranes with block ACE (DS Pharma Biomedical Co. Ltd, Japan) at ambient temperature for four hours, primary antibodies were added to the membranes and incubated overnight at 4 °C with primary antibodies for TGF-β1 (#SC-130348, Santa Cruz Biotechnology Inc., USA), total Smad2/3 (#3102, Cell Signaling Technology, USA), p-Smad2/3 (#8828, Cell Signaling Technology, USA), α-SMA (#NB300-978, Novus biologicals LLC., USA), and *β-actin* (#F1819, Santa Cruz Biotechnology, USA). The gout anti-rabbit immunoglobulin G (Novus Biologicals LLC., USA) secondary antibody coupled with horseradish peroxidase was used to detect the primary antibodies, and an enhanced chemiluminescence system (Clarity TM western ECL substrate, #170-5060, Bio-Rad Inc., CA, USA) was employed to display the results. A CCD-camera-based imager was used to record these signals. After normalizing target protein bands against *β-actin* bands (housekeeping protein), band density was estimated using ChemiDoc MP imaging system software (Bio-Rad Inc., Hercules, CA. USA). Both primary and secondary antibodies were employed at a dilution of 1:1000 in 5% BSA in PBST.

### Histopathological examination of kidney tissues

The kidney tissues were prepared for light microscopic study to create paraffin blocks by fixing them in 10% formaldehyde utilizing the tissue-embedding center (LEICA EG 1160, Wetzlar, Germany). Staining with hematoxylin and eosin (H&E) was performed on five-micrometer slices after being deparaffinized with xylene and rehydrated, as previously described^39^. Light microscopy and a digital slide scanner (Leica APERIO LV1 microscope) were used to examine the sections.

### Statistical analysis

Every statistic point was displayed as mean ± SEM. GraphPad Prism® 9 (GraphPad Software Inc., San Diago, USA) was used for a one-way ANOVA test. The Tukey-Kramar test was then used to examine the findings and determine whether significant variations between the groups existed. Any difference was deemed significant if its probability (p) value < 0.05.

### Ethical approval

The study was conducted in accordance with ARRIVE guidelines and executed in compliance with the Care and Use of Laboratory Animals standard operating procedures approved by the Research Ethics Committee, Minia University (Project code: MPEC (2301202)).

## Results

### Effect of nebivolol on kidney injury indices

**Figure 1:**
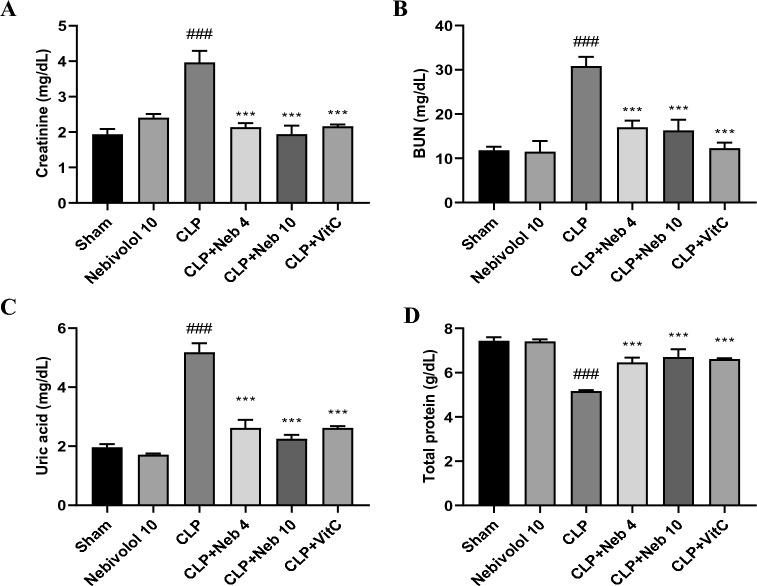
Effect of Nebivolol on serum creatinine (**A**), BUN (**B**), uric acid (**C**), and total protein (**D**) levels after induction of CLP in rats. Bars are represented as mean ± SEM. The Tukey-Kramar test was used to analyze the significant differences between groups after conducting a one-way ANOVA test, where ^###^p < 0.001, compared to the sham group, ***p < 0.001, compared to the CLP group.

Sepsis induced by CLP has a powerful injurious effect on the kidney, and its action has been affirmed by different markers^39^. CLP substantially (p < 0.001) increased serum Cr, BUN, and uric acid levels contrasted to sham group (Fig. 1). Additionally, it resulted in a substantial (p < 0.001) drop in the levels of serum total protein. Compared with the CLP group, the treated groups with Neb.4, Neb.10, or Vit C drastically (p < 0.001) reduced the changes in levels of Cr, BUN, uric acid, and total protein produced by CLP in the serum.

### Impact of nebivolol on renal oxidative stress

**Figure 2:**
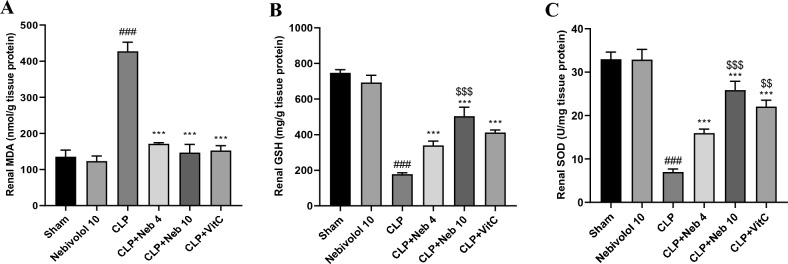
Effect of Nebivolol on oxidative stress status of kidney tissues, MDA content (**A**), GSH level (**B**), and SOD activity (**C**). Bars are represented as mean ± SEM. The Tukey-Kramar test was used to analyze the significant differences between groups after conducting a one-way ANOVA test, where ^###^p < 0.001, compared to the sham group, ***p < 0.001, compared to the CLP group, ^$$^p < 0.01 and ^$$$^p < 0.001, compared to CLP + Neb4 group.

As shown in Fig. 2, following induction of CLP, the kidney's MDA content was found to be notably (p < 0.001) higher than that in sham rats, whereas GSH level and SOD activity were significantly (p < 0.001) reduced. It's interesting to observe that when administrated in conjunction with CLP, Nebivolol (4 or 10 mg/kg) or vitamin C dramatically (p < 0.001) reduced the amount of MDA in kidney homogenate and increased renal GSH level and SOD activity in comparison to CLP rats. In addition, administration of 10 mg/kg Nebivolol in CLP + Neb10 group significantly increased the renal GSH level and SOD activity (p < 0.001 and p < 0.01, respectively) when compared with those of CLP + Neb4 group.

### Effect of nebivolol on kidney content of TNF*-*α*,* IL-6, IL-1β, IL-10, and caspase-3 proteins

Sepsis-induced inflammatory mediators, such as TNF-α, IL-6, and IL-1β, inhibited the protective anti-inflammatory cytokine IL-10. At the same time, sepsis induces apoptosis by overactivation of several apoptotic mediators, including caspase-3^40^.

**Figure 3:**
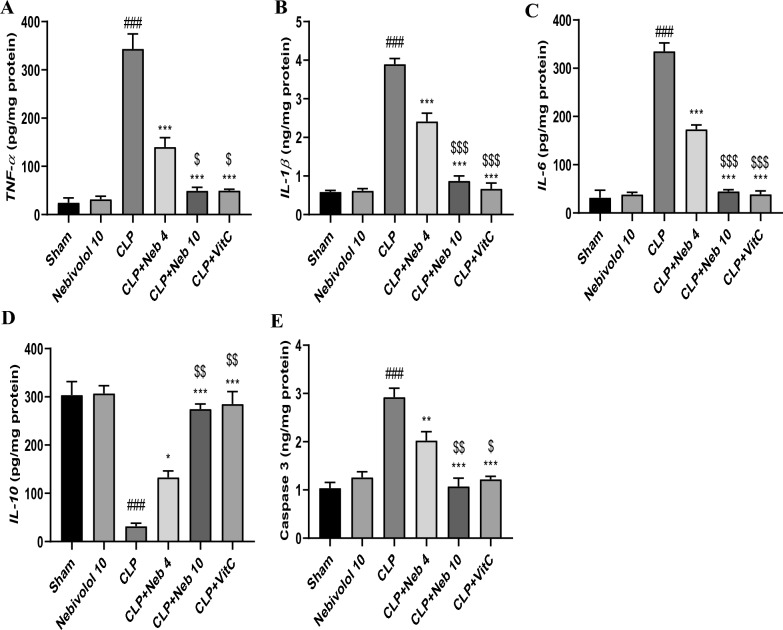
Effect of Nebivolol on the pro-inflammatory cytokines, TNF-α (**A**), IL-β (**B**), IL-6 (**C**), the anti-inflammatory cytokine IL-10 (**D**), and the pro-apoptotic mediator Caspase-3 (**E**) in kidney tissues. Bars are represented as mean ± SEM. The Tukey-Kramar test was used to analyze the significant differences between groups after conducting a one-way ANOVA test, where ^###^p < 0.001, compared to the sham group, *p < 0.05,**p < 0.01, and ***p < 0.001, compared to CLP group, ^$^p < 0.05, ^$$^p < 0.01, and ^$$$^p < 0.001, compared to CLP + Neb4 group.

The present study utilized ELISA to assess the protein levels of the pro-inflammatory (TNF-α, IL-6, and IL-1β), the anti-inflammatory (IL-10) cytokines, and the pro-apoptotic mediator caspase-3 in CLP and treated groups. As illustrated in Fig. 3, CLP induction, compared to sham, drastically (p < 0.001) elevated the renal protein amounts of TNF-α, IL-6, IL-1β, and caspase-3. Furthermore, it markedly (p < 0.001) reduced the IL-10 level in kidney tissues. The administration of drugs of interest (Nebivolol (4 or 10 mg/kg) or Vit C) with CLP surgery significantly reduced the pro-inflammatory mediators and the caspase-3 levels while significantly raising the anti-inflammatory IL-10 level in comparison to CLP rats.

### Effect of nebivolol on *Bax, Bcl-2*, p53, and NF-κB genes expression

**Figure 4:**
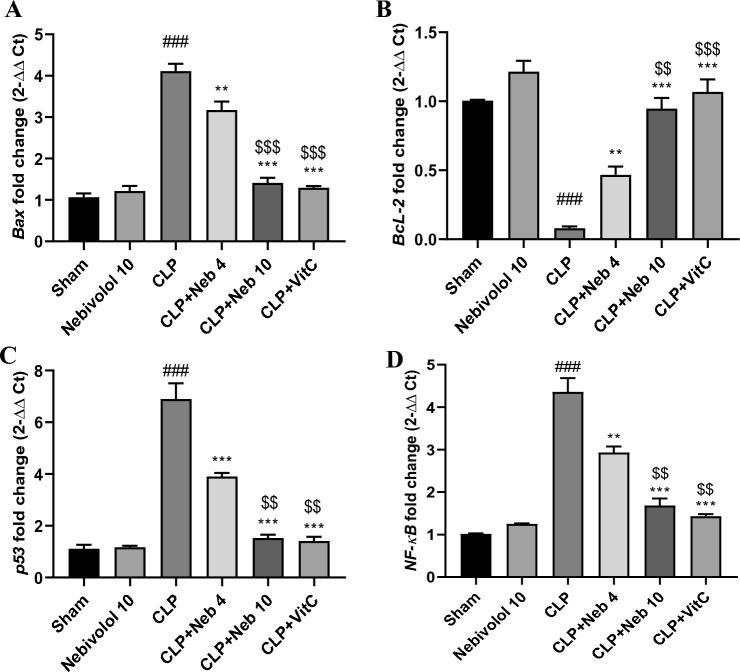
Effect of Nebivolol on *Bax* (**A**), *Bcl-2* (**B**), *p53* (**C**), and *NF-kB* (**D**) genes expression in kidney tissues. Bars are represented as mean ± SEM. The Tukey-Kramar test was used to analyze the significant differences between groups after conducting a one-way ANOVA test, where ^###^p < 0.001, compared to the sham group, **p < 0.01, and ***p < 0.001, compared to the CLP group, ^$$^p < 0.01, and ^$$$^p < 0.001, compared to CLP + Neb4 group.

Overactivation of pro-fibrotic and pro-apoptotic agents, such as *Bax*, p53, and NF-κB, and suppression of the anti-apoptotic mediator *Bcl-2* in the kidney contributes to its apoptosis and fibrosis. Therefore, we measured the impact of Nebivolol on mRNA levels of *Bax, Bcl-2*, p53, and NF-κB to estimate its potency at different dosages as a protective molecule against kidney apoptosis and fibrosis induced by CLP. As Fig. 4 illustrates, *Bax*, *p53*, and *NF-κB* genes all exhibit markedly (p < 0.001) increased expression in response to CLP. In addition, it notably (p < 0.001) decreased *Bcl-2* gene expression contrasted with sham rats. However, both Nebivolol doses and Vitamin C demonstrated a significant reduction in the expression of the *Bax*, p53, and NF-κB genes while boosting the expression of the *Bcl-2* gene compared to the CLP group.

### Effect of nebivolol on TGF-β1, p-Smad2/3, and α-SMA proteins expression

**Figure 5:**
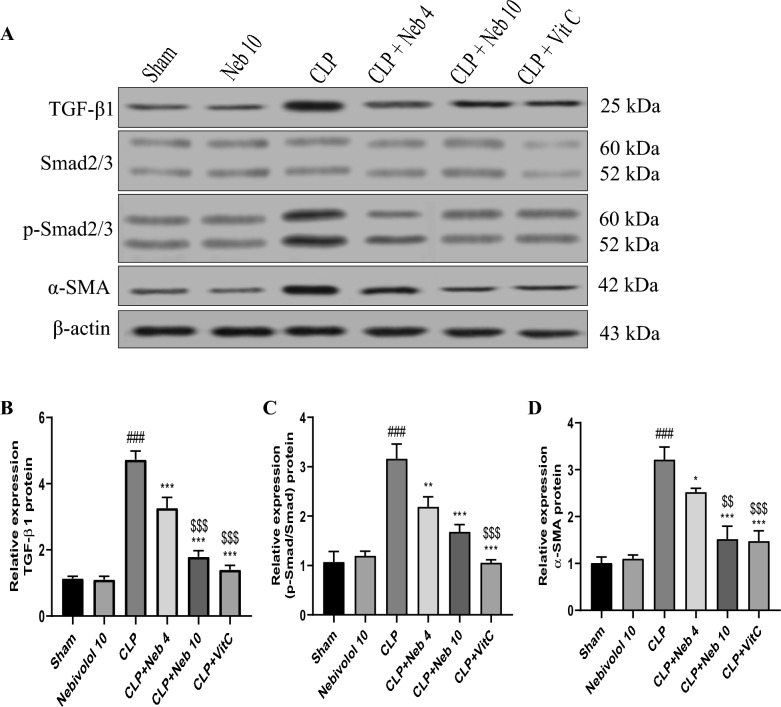
Effect of Nebivolol on TGF-β1, p-Smad2/3, and α-SMA proteins expression. (**A**) representative western blot membranes of TGF-β1, Smad2/3, p-Smad2/3, α-SMA, and β-actin proteins for all studied groups. (**B**–**D**) Expressions of TGF-β1, p-Smad2/3/t-Smad2/3, and α-SMA proteins were represented densitometrically using bands in (**A**). Bars are represented as mean ± SEM. The Tukey-Kramar test was used to analyze the significant differences between groups after conducting a one-way ANOVA test, where ^###^p < 0.001, compared to the sham group, *p < 0.05,**p < 0.01, and ***p < 0.001, compared to CLP group, ^$$^p < 0.01, and ^$$$^p < 0.001, compared to CLP + Neb4 group.

To check the protective impact of Nebivolol on kidney fibrosis, we assessed the blocking effect of Nebivolol on TGF-β1/Smad/α-SMA signaling cascade triggered by CLP via western blotting. Figure 5 illustrates after normalization of the intensities of targeted bands to β-actin, CLP exhibited an enormously (p < 0.001) enhanced up-regulation of TGF-β1, p/t-Smad2/3, and α-SMA proteins in the kidneys as compared to sham rats. Treated groups with Nebivolol (4 or 10 mg/kg) or Vit C showed a significant down-regulation of all proteins compared to septic rats in the CLP group.

### Effect of nebivolol on histological changes of kidney tissues

**Figure 6:**
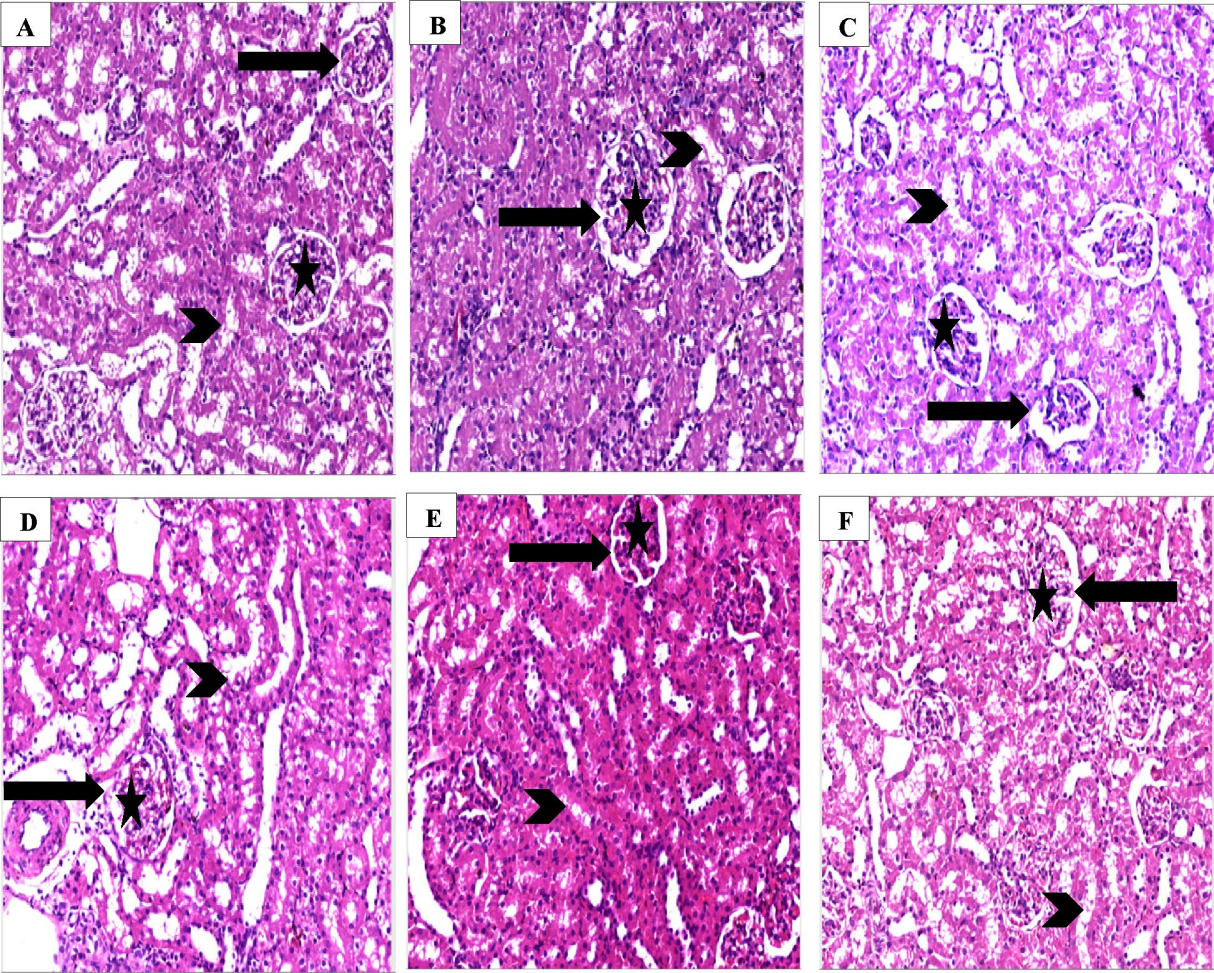
Effect of Nebivolol on kidney histological changes. The figure represents the photomicrographs of rat renal cortex (H&E staining, magnification; 400 ×). (**A**) The sham group showed normal glomeruli (star), Bowman's capsule (black arrow) tubules (arrowhead), (**B**) Nebivolol 10 group showing normal structure kidney, (**C**) CLP group showing collapsed renal glomeruli with widened Bowman's capsule and degenerated tubule, (**D**) CLP/Nebivolol 4 group showing a few renal glomeruli with glomerular capillary collapse and widened Bowman's capsule. Tubules showed cloudy swelling and cytoplasmic vacuolations, (**E**) CLP/Nebivolol 10 group showed near-normal renal glomeruli, mild tubular cytoplasmic vacuolation, and (**F**) CLP/vitamin C group showed mild tubular cytoplasmic vacuolation, and degeneration.

When compared with kidneys of the sham group rats that displayed standard renal tubules, glomeruli, and vasculature, the kidneys of the CLP group rats displayed collapsed glomeruli and dilated Bowman's capsule, degraded tubules, and regions of diffuse hemorrhage. The rats of CLP/Nebivolol 4 group revealed a few renal glomeruli with glomerular capillary collapse and expanded Bowman’s capsule. Cytoplasmic vacuolations and fuzzy edema were seen in the tubules. In addition, the CLP/Nebivolol 10 group showed near-normal renal glomeruli, minor tubular cytoplasmic vacuolation, and healthy vasculature. However, the CLP/Vitamin C group showed mild tubular cytoplasmic vacuolation and degeneration, as illustrated in Fig. 6 and Table 2.

**Table 2:** Renal tissue scoring representation throughout the different groups.

	Sham	Nebivolol 10	CLP	CLP + Nebivolol 4	CLP + Nebivolol 10	CLP + Vit C
Interstitial						
Hemorrhage	0	0	2	1	0	0
Edema	0	0	2	1	0	1
Vascular congestion	0	0	3	2	1	1
Renal corpuscle						
G. vacuolation collapse	0	0	3	2	0	1
Shrinkage/distorted corpuscle	0	0	3	2	1	1
Widening BC space	0	0	3	2	0	0
Renal tubules						
Cellular apoptosis	0	0	2	2	1	1
Cellular vacuolation	0	0	3	2	1	0
Lumen widening and distortion	0	0	2	2	1	1
Casts	0	0	1	0	0	0

0 represents no change, 1 denotes a minor change, 2 is a moderate change, and 3 denotes a severe change.

## Discussion

Sepsis, a clinical syndrome of having an extreme systemic inflammatory reaction syndrome (SIRS) to the pathogen, is considered the most frequent causal factor concerning AKI progression. AKI is strongly correlated with weak prognostic consequences involving CKD, heart events, and even demise. While the exact etiology of S-AKI is unknown, sepsis is thought to be responsible for 45–70% of AKI episodes in patients with serious conditions^6,7^. Australia had approximately 11.7% incidence of S-AKI, and the frequency in Beijing was roughly 48.1%, with a 55% overall fatality rate^41^.

It is difficult to improve outcomes in S-AKI because individuals may exhibit clinical or subclinical AKI, and AKI linked to sepsis may manifest with distinct phenotypes and prognoses^42^. Several aspects of S-AKI are poorly understood, including its epidemiology, pathophysiology, biomarker’s role in risk assessment and treatment protocols, and the impact of new treatments on patient outcomes.

Rats' CLP operation was shown to cause sepsis with additional kidney damage and renal failure, mirroring human illness^43^. Various approaches, using synthetic or natural candidates, have been applied to relief the renal toxicity generated from CLP-induced sepsis by targeting oxidative stress, apoptosis, and fibrosis in damaged kidney tissues^43–47^. Nebivolol administration was recently reported to diminish ischemia–reperfusion, contrast media, and drug-induced kidney failure through its anti-oxidant and anti-inflammatory effects^48–50^. To the best of our information, no previous research has looked into the potential preventive role of Nebivolol against sepsis-evoked organ damage. This study is the first one to assess the possible potential signaling mechanism of Nebivolol and its protective effect on kidney fibrosis resulted from CLP-induced sepsis.

For the first time in CLP-induced nephrotoxicity, the finding of this study showed that pretreatment with Nebivolol, in a dose-dependent manner, reduced the elevated levels of uric acid, Cr, and BUN serum levels and increased the level of serum total protein. Moreover, Nebivolol reduced renal MDA content, protein levels of TNF-α, IL-1β, IL-6, and caspase-3, mRNA expression of *Bax*, *p53*, and *NF-*κ*B*, and the protein expression of TGF-β1, p-Smad2/3, and α-SMA. In addition, it increased SOD activity, GSH and IL-10 levels, and up-regulated the *Bcl-2* mRNA renal expression.

In the present study, induction of sepsis through CLP surgery developed a marked deterioration in kidney function through increasing the serum levels of its markers, including Cr, BUN and uric acid. In addition, a notable reduction in the total protein content was observed. These biochemical results matched the histological alterations displayed in the septic rats. The pathophysiology of the harmful effect of sepsis on the kidney involves several pathways, including the aggravation of oxidative stress, inflammation, and immune function deterioration^51^. Sepsis-related oxidative stress stimulates the body's adaptive response to tissue hypoxia, apoptosis and further fibrosis^52^. Therefore, the lipid peroxidation (oxidative stress) marker, MDA, as well as the anti-oxidant markers, including SOD and GSH were assessed in kidney homogenates. In this study, the renal tissues of CLP-intoxicated rats had a significant increase MDA content. Additionally, there was a marked decrease in their SOD activity and GSH level in contrast with the normal rats. It disclaimed that an elevation in ROS generates sepsis-persuaded kidney damage by decreasing the host’s anti-oxidant defense mechanisms, leading to cell damage^51,53^.

Numerous pro-inflammatory and pro-fibrotic signaling networks, including TGF-β1, NF-κB, p53, and MAPK family members, are activated more strongly by ROS and oxidative stress^53–56^. It was implicated that TGF-β1 has a crucial function in generating EMT and is the driver of kidney fibrogenesis through TGF-β1/Smad/p53 signaling pathways^57–60^. During sepsis, active TGF-β1 is released and attached to its receptor (TGFβI/II) resulting in the phosphorylation and activation for Smad2/3, which binds with Smad4, leading to the formation of an active complex of Smad2/3/4. At the same time, TGF-β1 can stimulate the non-Smad pathway, leading to activation and phosphorylation of p53-dependant tubular epithelial G2/M arrest^61^, leading to the assembly of p53-Smad complex. This complex is translocated in the nucleus and binds to target genes responsible for the induction of EMT, inflammation and renal fibrosis^57,62,63^. α-SMA is a positive EMT marker for myofibroblast, as it activates fibroblast, which is deemed a central effector cell of renal injury and fibrogenesis^64^. Additionally, TGF-β1 can induce TAK1/IKK/IkBα phosphorylation and subsequently stimulate the phosphorylation of the downstream NF-κB inflammatory transcriptional factor, which translocates and accumulates in the nucleus to facilitate the transcription of multiple inflammatory cytokines, including TNF-α, IL-1β and IL-6, while also diminishing the anti-inflammatory cytokine, IL-10^57,65,66^. This study revealed that CLP-induced sepsis can evoke renal fibrosis through EMT activation, affirmed by the elevation of α-SMA marker in septic rats contrasted to the sham group. We demonstrated that CLP stimulates EMT by dramatically upregulating the protein expression of α-SMA. Furthermore, the protein expressions of renal TGF-β1, p-Smad2/3, and also the expression of *p53* and *NF-kB* genes were assessed in this study to illuminate the probable signaling pathway by which CLP can stimulate EMT, renal fibrosis, and inflammation. Our results supported that CLP resulted in an increase in TGF-β1 and Smad2/3 proteins expression, as well as an increase in the expression of *p53* and *NF-kB* genes, which further up-regulated the inflammatory mediators TNF-α, IL-6, and IL-1β and inhibited the anti-inflammatory cytokine, IL-10. In such a manner, the present study illustrated that sepsis affects the onset of EMT, renal fibrosis, and inflammation by modulating TGF-β1/Smad/p53 and TGF-β1/NF-κB signaling cascades.

The Bcl-2 family has a significant role in transducing apoptotic signals within cells. Apoptosis is directly linked to the pro- and anti-apoptotic *Bax* and *Bcl-2* genes; the ratio of *Bax* to *Bcl-2* expression is a cell death button that controls a cell's sensitivity to an apoptotic stimulus, determining whether it will proliferate or die^67^. The overexpression of *Bax* and diminished *Bcl-2* leads to the release of cytochrome c in the cytoplasm, which triggers caspases (caspase 3/7) and results in irreversible apoptosis and cell death^68^. It was found that activation of TGF-β1/NF-κB pathway triggered apoptosis by targeting the mitochondrial pathway. That was accomplished by lowering the level of *Bcl-2* and raising the amounts of *Bax* and caspase-3. Consistently, kidney tissues of septic rats in this study showed an elevation in the expressions of *Bax* gene and Caspase-3 protein and diminished expression of *Bcl-2* gene concurrently with the previous studies^69,70^, suggesting significant apoptosis in these rats.

ROS is known to contribute to the renal disease onset and progression through the induction of TGF-β1. Therefore, targeting ROS and TGF-β1 expression may be valuable in treating renal fibrosis^14,71^. As a result, we assumed that candidates with an anti-oxidant perspective or that target TGF-β1 may inhibit sepsis-evoked kidney fibrosis. Recently, Nebivolol was revealed to inhibit inflammation in ischemia–reperfusion and drug-induced toxicity in various organs due to its potentiality to target and reduce oxidative stress^35,36,48–50,72–74^. These discoveries suggest that Nebivolol may protect kidneys against CLP-evoked sepsis as demonstrated in this study.

Nebivolol dramatically reduced kidney damage and restored renal function in the current trial by significantly lowering uric acid, creatinine, and BUN serum levels while powerfully restoring serum total protein levels. In addition, the histological examination, which validated the recovery in all morphological alterations of kidney construction, supported these findings. According to our study, administering Nebivolol to septic rats results in a concentration-dependent reduction in lipid peroxidation, MDA content, a significant increase in anti-oxidant status, GSH levels and SOD activity, and a decrease in renal damage brought on by oxidative stress. These results are consistent with those of Gandhi et al., who found that Nebivolol, in injured kidneys by ischemia–reperfusion, exhibits anti-oxidant activity through the reduction of MDA content as well as the elevation of SOD activity and GSH levels^75^.

Moreover, for CLP-intoxicated rats, treatment with Nebivolol significantly suppressed the renal upsurge in protein expression of TGF-β1, p-Smad2/3, and α-SMA, as well as reduced the gene expression of *p53*, suggesting that Nebivolol could inhibit renal fibrosis in septic rats through the attenuation of TGF-β1/Smad2/3/p53 signaling pathway which stimulates EMT process. Additionally, in the present study, Nebivolol was found to suppress the gene expression of *NF-*κ*B* and further its downstream inflammatory negotiators, TNF-α, IL-1β, IL-6, and upregulate the anti-inflammatory cytokine IL-10, indicating that Nebivolol has anti-inflammatory activity. Furthermore, it implied an anti-apoptotic effect by significantly suppressing the expession of *Bax* gene and caspase-3 protein and enhancing the expression of *Bcl-2* gene.

Thus, the current data clearly showed the nephroprotective effect of Nebivolol, which might be a novel therapeutic drug for ameliorating sepsis-evoked kidney dysfunction through targeting oxidative stress and TGF-β1 canonical and non-canonical pathways.

## Conclusions

This study, for the first time, demonstrated that Nebivolol can successfully exert a promising protective potentiality against kidney dysfunction resulted from CLP-induced sepsis in rats. This protective effect was associated with suppressing the Cr, BUN, and serum uric acid levels, and increasing the serum total protein level. Furthermore, it alleviated oxidative stress and inflammatory cytokines by reducing MDA, TNF-α, IL-6, and IL-1β levels. On the other hand, it increased the antioxidant protective mediators, including SOD activity, GSH and IL-10 levels.

Furthermore, it attenuated the kidney fibrosis by suppressing the protein levels of TGF-β1, p-Smad2/3, and α-SMA and the expression level of *p53* gene. Additionally, Nebivolol inhibited kidney apoptosis as it repressed the expressions of *NF-*κ*B*, *Bax* genes and caspase-3 protein levels, enhancing the expression of the anti-apoptotic *Bcl-2* gene. Although further studies are needed to assess its efficacy and safety, our study concluded that Nebivolol can be a novel candidate for mitigating the sepsis-induced kidney dysfunction and its protective potentiality is achieved by inhibiting oxidative stress, inflammation, apoptosis, and fibrosis through targeting TGF-β1/Smad2/3/p53 and TGF-β1/NF-κB signaling cascades (Supplementary Information S1).

### Supplementary Information

Supplementary Information.

## Data Availability

Data is provided within the manuscript or supplementary information files.

## References

[1] Nedeva C, Menassa J, Duan M, Liu C, Doerflinger M, Kueh AJ, Herold MJ, Fonseka P, Phan TK, Faou P, Rajapaksha H, Chen W, Hulett MD, Puthalakath H (2020). TREML4 receptor regulates inflammation and innate immune cell death during polymicrobial sepsis. Nat. Immunol..

[2] Singer M, Deutschman CS, Seymour CW, Shankar-Hari M, Annane D, Bauer M, Bellomo R, Bernard GR, Chiche J-D, Coopersmith CM (2016). The third international consensus definitions for sepsis and septic shock (Sepsis-3). JAMA.

[3] Srzić I, Nesek Adam V, Tunjić Pejak D (2022). Sepsis definition: What’s new in the treatment guidelines. Acta Clin. Croat..

[4] Rudd KE, Johnson SC, Agesa KM, Shackelford KA, Tsoi D, Kievlan DR, Colombara DV, Ikuta KS, Kissoon N, Finfer S, Fleischmann-Struzek C, Machado FR, Reinhart KK, Rowan K, Seymour CW, Watson RS, West TE, Marinho F, Hay SI, Lozano R, Lopez AD, Angus DC, Murray CJL, Naghavi M (2020). Global, regional, and national sepsis incidence and mortality, 1990–2017: Analysis for the Global Burden of Disease Study. Lancet.

[5] Shappell CN, Klompas M, Rhee C (2020). Surveillance strategies for tracking sepsis incidence and outcomes. J. Infect. Dis..

[6] Hoste EA, Bagshaw SM, Bellomo R, Cely CM, Colman R, Cruz DN, Edipidis K, Forni LG, Gomersall CD, Govil D, Honoré PM, Joannes-Boyau O, Joannidis M, Korhonen AM, Lavrentieva A, Mehta RL, Palevsky P, Roessler E, Ronco C, Uchino S, Vazquez JA, Vidal Andrade E, Webb S, Kellum JA (2015). Epidemiology of acute kidney injury in critically ill patients: The multinational AKI-EPI study. Intensive Care Med..

[7] Uchino S, Kellum JA, Bellomo R, Doig GS, Morimatsu H, Morgera S, Schetz M, Tan I, Bouman C, Macedo E, Gibney N, Tolwani A, Ronco C (2005). Acute renal failure in critically ill patients: A multinational, multicenter study. JAMA.

[8] Thakar CV, Christianson A, Freyberg R, Almenoff P, Render ML (2009). Incidence and outcomes of acute kidney injury in intensive care units: A Veterans Administration study. Crit. Care Med..

[9] Ruiz-Ortega M, Rayego-Mateos S, Lamas S, Ortiz A, Rodrigues-Diez RR (2020). Targeting the progression of chronic kidney disease. Nat. Rev. Nephrol..

[10] Wang W, Koka V, Lan HY (2005). Transforming growth factor-beta and Smad signalling in kidney diseases. Nephrology (Carlton).

[11] Fathy M, Okabe M, Saad Eldien HM, Yoshida T (2020). AT-MSCs antifibrotic activity is improved by eugenol through modulation of TGF-beta/smad signaling pathway in rats. Molecules.

[12] Hewlett JC, Kropski JA, Blackwell TS (2018). Idiopathic pulmonary fibrosis: Epithelial-mesenchymal interactions and emerging therapeutic targets. Matrix Biol..

[13] Alaaeldin R, Abuo-Rahma GEA, Zhao QL, Fathy M (2021). Modulation of apoptosis and epithelial-mesenchymal transition E-cadherin/TGF-beta/Snail/TWIST pathways by a new ciprofloxacin chalcone in breast cancer cells. Anticancer Res..

[14] Isaka Y (2018). Targeting TGF-β signaling in kidney fibrosis. Int. J. Mol. Sci..

[15] Németh Á, Mózes MM, Calvier L, Hansmann G, Kökény G (2019). The PPARγ agonist pioglitazone prevents TGF-β induced renal fibrosis by repressing EGR-1 and STAT3. BMC Nephrol..

[16] Bekhit AA, Beshay ON, Fawzy MA, Abdel-Hafez SMN (2023). Curative effect of AD-MSCs against cisplatin-induced hepatotoxicity in rats is potentiated by azilsartan: targeting oxidative stress, MAPK, and apoptosis signaling pathways. Stem Cells Int..

[17] Fawzy MA, Maher SA, Bakkar SM, El-Rehany MA, Fathy M (2021). Pantoprazole attenuates MAPK (ERK1/2, JNK, p38)-NF-kappaB and apoptosis signaling pathways after renal ischemia/reperfusion injury in rats. Int. J. Mol. Sci..

[18] Fawzy MA, Nasr G, Ali FEM, Fathy M (2023). Quercetin potentiates the hepatoprotective effect of sildenafil and/or pentoxifylline against intrahepatic cholestasis: Role of Nrf2/ARE, TLR4/NF-κB, and NLRP3/IL-1β signaling pathways. Life Sci..

[19] Abdellatef AA, Fathy M, Mohammed AEI, Bakr MSA, Ahmed AH, Abbass HS, El-Desoky AH, Morita H, Nikaido T, Hayakawa Y (2021). Inhibition of cell-intrinsic NF-kappaB activity and metastatic abilities of breast cancer by aloe-emodin and emodic-acid isolated from Asphodelus microcarpus. J. Nat. Med..

[20] Alaaeldin R, Hassan HA, Abdel-Rahman IM, Mohyeldin RH, Youssef N, Allam AE, Abdelwahab SF, Zhao QL, Fathy M (2022). A new EGFR inhibitor from ficus benghalensis exerted potential anti-inflammatory activity via Akt/PI3K pathway inhibition. Curr. Issues Mol. Biol..

[21] Fawzy MA, Maher SA, El-Rehany MA, Welson NN, Albezrah NKA, Batiha GE-S, Fathy M (2022). Vincamine modulates the effect of pantoprazole in renal ischemia/reperfusion injury by attenuating MAPK and apoptosis signaling pathways. Molecules.

[22] Fathy M, Sun S, Zhao Q-L, Abdel-Aziz M, Abuo-Rahma GE-DA, Awale S, Nikaido T (2020). A new ciprofloxacin-derivative inhibits proliferation and suppresses the migration ability of HeLa cells. Anticancer Res..

[23] Mohyeldin RH, Alaaeldin R, Sharata EE, Attya ME, Elhamadany EY, Fathy M (2023). LCZ696 attenuates sepsis-induced liver dysfunction in rats; the role of oxidative stress, apoptosis, and JNK1/2-P38 signaling pathways. Life Sci..

[24] Shytaj IL, Fares M, Gallucci L, Lucic B, Tolba MM, Zimmermann L, Adler JM, Xing N, Bushe J, Gruber AD, Ambiel I, Taha Ayoub A, Cortese M, Neufeldt CJ, Stolp B, Sobhy MH, Fathy M, Zhao M, Laketa V, Diaz RS, Sutton RE, Chlanda P, Boulant S, Bartenschlager R, Stanifer ML, Fackler OT, Trimpert J, Savarino A, Lusic M (2022). The FDA-approved drug cobicistat synergizes with remdesivir to inhibit SARS-CoV-2 replication in vitro and decreases viral titers and disease progression in syrian hamsters. mBiol..

[25] Alaaeldin R, Bakkar SM, Mohyeldin RH, Ali FEM, Abdel-Maqsoud NMR, Fathy M (2023). Azilsartan modulates HMGB1/NF-kappa;B/p38/ERK1/2/JNK and apoptosis pathways during renal ischemia reperfusion injury. Cells.

[26] Sabra RT, Abdellatef AA, Abdel-Sattar E, Fathy M, Meselhy MR, Hayakawa Y (2022). Russelioside A, a pregnane glycoside from *Caralluma tuberculate*, inhibits cell-intrinsic NF-κB activity and metastatic ability of breast cancer cells. Biol. Pharmaceut. Bull..

[27] de Groot AA, Mathy MJ, van Zwieten PA, Peters SL (2004). Antioxidant activity of nebivolol in the rat aorta. J. Cardiovasc. Pharmacol..

[28] Oelze M, Daiber A, Brandes RP, Hortmann M, Wenzel P, Hink U, Schulz E, Mollnau H, von Sandersleben A, Kleschyov AL, Mülsch A, Li H, Förstermann U, Münzel T (2006). Nebivolol inhibits superoxide formation by NADPH oxidase and endothelial dysfunction in angiotensin II-treated rats. Hypertension.

[29] Wolf SC, Sauter G, Jobst J, Kempf VA, Risler T, Brehm BR (2008). Major differences in gene expression in human coronary smooth muscle cells after nebivolol or metoprolol treatment. Int. J. Cardiol..

[30] Mercanoglu G, Safran N, Gungor M, Pamukcu B, Uzun H, Sezgin C, Mercanoglu F, Fici F (2008). The effects of nebivolol on apoptosis in a rat infarct model. Circ. J..

[31] Fink MP (2014). Animal models of sepsis. Virulence.

[32] Bhatia A, Saikia PP, Dkhar B, Pyngrope H (2022). Anesthesia protocol for ear surgery in Wistar rats (animal research). Anim. Mod. Exp. Med..

[33] Abdallah O, Sharaf Eldin A (2016). Nebivolol ameliorates indomethacin-induced gastric ulcer in adult albino rats: Role of inducible nitric oxide synthase. Egypt. J. For. Sci. Appl. Toxicol..

[34] Mohamed EA, Kassem HH (2018). Protective effect of nebivolol on doxorubicin-induced cardiotoxicity in rats. Arch. Med. Sci..

[35] Said ES, Mohammed AH, Ali HM, Babiker AY, Alnughaymishi R, Althaqeel NZ, Ahmed AS (2022). Evaluation of hepatoprotective effect of nebivolol and sodium copper chlorophyllin on CCL4-induced hepatotoxicity in mice. Eur. Rev. Med. Pharmacol. Sci..

[36] Li X, Li Q, Wu L, Wang Y (2023). Nebivolol alleviates vascular endothelial insulin resistance by inhibiting endoplasmic reticulum stress. Int. Heart J..

[37] Naeem AG, El-Naga RN, Michel HE (2022). Nebivolol elicits a neuroprotective effect in the cuprizone model of multiple sclerosis in mice: emphasis on M1/M2 polarization and inhibition of NLRP3 inflammasome activation. Inflammopharmacology.

[38] Zhang N, Zhao W, Hu ZJ, Ge SM, Huo Y, Liu LX, Gao BL (2021). Protective effects and mechanisms of high-dose vitamin C on sepsis-associated cognitive impairment in rats. Sci. Rep..

[39] Peerapornratana S, Manrique-Caballero CL, Gómez H, Kellum JA (2019). Acute kidney injury from sepsis: Current concepts, epidemiology, pathophysiology, prevention and treatment. Kidney Int..

[40] Chaudhry H, Zhou J, Zhong Y (2013). Role of cytokines as a double-edged sword in sepsis. In Vivo.

[41] Wang H, Ji X, Wang AY, Wu PK, Liu Z, Dong L, Liu J, Duan M (2021). Epidemiology of sepsis-associated acute kidney injury in Beijing, China: A descriptive analysis. Int. J. Gen. Med..

[42] Wiersema R, Jukarainen S, Vaara ST, Poukkanen M, Lakkisto P, Wong H, Linder A, van der Horst ICC, Pettilä V (2020). Two subphenotypes of septic acute kidney injury are associated with different 90-day mortality and renal recovery. Crit. Care.

[43] Borges-Rodriguez M, Shields CA, Travis OK, Tramel RW, Baik CH, Giachelli CA, Tardo GA, Williams JM, Cornelius DC (2021). Platelet inhibition prevents NLRP3 inflammasome activation and sepsis-induced kidney injury. Int. J. Mol. Sci..

[44] Jabber H, Mohammed B, Hadi NR (2023). Investigating the renoprotective effect of C21 in male mice with sepsis via modulation of p-AKT/PI3K expression. J. Med. Life.

[45] Chen X, Tong H, Chen Y, Chen C, Ye J, Mo Q, Zhao G, Hong G, Zheng C, Lu Z (2018). Klotho ameliorates sepsis-induced acute kidney injury but is irrelevant to autophagy. Oncol. Targets Ther..

[46] Guo L-P, Liu S-X, Yang Q, Liu H-Y, Xu L-L, Hao Y-H (2020). Effect of thymoquinone on acute kidney injury induced by sepsis in BALB/c mice. BioMed. Res. Int..

[47] Abdelnaser M, Alaaeldin R, Attya ME, Fathy M (2024). Modulating Nrf-2/HO-1, apoptosis and oxidative stress signaling pathways by gabapentin ameliorates sepsis-induced acute kidney injury. Naunyn Schmiedebergs Arch. Pharmacol..

[48] Cavdar Z, Kocak A, Ural C, Afagh A, Ersan S, Ozbal S, Tatli M, Celik A, Arslan S, Cavdar C (2023). Role of p38 MAPK, Akt and NFκB in renoprotective effects of nebivolol on renal ischemia-reperfusion injury in rats. Biotech. Histochem..

[49] Nasr AM, Rezq S, Shaheen A, Elshazly SM (2020). Renal protective effect of nebivolol in rat models of acute renal injury: Role of sodium glucose co-transporter 2. Pharmacol. Rep..

[50] Wanas H, El-Shabrawy M, Mishriki A, Attia H, Emam M, Aboulhoda BE (2021). Nebivolol protects against cyclophosphamide-induced nephrotoxicity through modulation of oxidative stress, inflammation, and apoptosis. Clin. Exp. Pharmacol. Physiol..

[51] Ow CPC, Trask-Marino A, Betrie AH, Evans RG, May CN, Lankadeva YR (2021). Targeting oxidative stress in septic acute kidney injury: From theory to practice. J. Clin. Med..

[52] Dröge W (2002). Free radicals in the physiological control of cell function. Physiol. Rev..

[53] Biswal S, Remick DG (2007). Sepsis: Redox mechanisms and therapeutic opportunities. Antioxid. Redox. Signal.

[54] Son Y, Cheong YK, Kim NH, Chung HT, Kang DG (2011). Mitogen-activated protein kinases and reactive oxygen species: How can ROS activate MAPK pathways?. J. Signal Transduct..

[55] Abdelnaser M, Alaaeldin R, Attya ME, Fathy M (2023). Hepatoprotective potential of gabapentin in cecal ligation and puncture-induced sepsis; targeting oxidative stress, apoptosis, and NF-kB/MAPK signaling pathways. Life Sci..

[56] Alaaeldin R, Mohyeldin RH, Bekhit AA, Gomaa W, Zhao QL, Fathy M (2023). Vincamine ameliorates epithelial-mesenchymal transition in bleomycin-induced pulmonary fibrosis in rats; targeting TGF-beta/MAPK/snai1 pathway. Molecules.

[57] Gu Y-Y, Liu X-S, Huang X-R, Yu X-Q, Lan H-Y (2020). Diverse role of TGF-β in kidney disease. Front. Cell Dev. Biol..

[58] Fawzy MA, Beshay ON, Bekhit AA, Abdel-Hafez SMN, Batiha GE-S, Bin Jardan YA, Fathy M (2023). Nephroprotective effect of AT-MSCs against cisplatin-induced EMT is improved by azilsartan via attenuating oxidative stress and TGF-β/Smad signaling. Biomed. Pharmacother..

[59] Sarkaki A, Badavi M, Nejaddehbashi F, Hajipour S, Basir Z, Amini N (2023). The renoprotective effects of hesperidin on kidney injury induced by exposure to severe chronic dust storm particulate matter through inhibiting the Smads/TGF-beta1 signaling in rat. Naunyn Schmiedebergs Arch. Pharmacol..

[60] Amini N, Sarkaki A, Dianat M, Mard SA, Ahangarpour A, Badavi M (2019). The renoprotective effects of naringin and trimetazidine on renal ischemia/reperfusion injury in rats through inhibition of apoptosis and downregulation of micoRNA-10a. Biomed. Pharmacother.

[61] Yang L, Besschetnova TY, Brooks CR, Shah JV, Bonventre JV (2010). Epithelial cell cycle arrest in G2/M mediates kidney fibrosis after injury. Nat. Med..

[62] Saitoh M (2015). Epithelial-mesenchymal transition is regulated at post-transcriptional levels by transforming growth factor-β signaling during tumor progression. Cancer Sci..

[63] Samarakoon R, Dobberfuhl AD, Cooley C, Overstreet JM, Patel S, Goldschmeding R, Meldrum KK, Higgins PJ (2013). Induction of renal fibrotic genes by TGF-β1 requires EGFR activation, p53 and reactive oxygen species. Cell Signal.

[64] Huang R, Fu P, Ma L (2023). Kidney fibrosis: From mechanisms to therapeutic medicines. Signal Transd. Target. Ther..

[65] Freudlsperger C, Bian Y, Contag Wise S, Burnett J, Coupar J, Yang X, Chen Z, Van Waes C (2013). TGF-β and NF-κB signal pathway cross-talk is mediated through TAK1 and SMAD7 in a subset of head and neck cancers. Oncogene.

[66] Alaaeldin R, Eisa YA, El-Rehany MA, Fathy M (2024). Vincamine alleviates intrahepatic cholestasis in rats through modulation of NF-kB/PDGF/klf6/PPARgamma and PI3K/Akt pathways. Naunyn Schmiedebergs Arch. Pharmacol..

[67] Vaskivuo TE, Stenbäck F, Tapanainen JS (2002). Apoptosis and apoptosis-related factors Bcl-2, Bax, tumor necrosis factor-alpha, and NF-kappaB in human endometrial hyperplasia and carcinoma. Cancer.

[68] Vince JE, De Nardo D, Gao W, Vince AJ, Hall C, McArthur K, Simpson D, Vijayaraj S, Lindqvist LM, Bouillet P, Rizzacasa MA, Man SM, Silke J, Masters SL, Lessene G, Huang DCS, Gray DHD, Kile BT, Shao F, Lawlor KE (2018). The mitochondrial apoptotic effectors BAX/BAK activate caspase-3 and -7 to trigger NLRP3 inflammasome and caspase-8 driven IL-1β activation. Cell Rep..

[69] Bedirli N, Bagriacik EU, Yilmaz G, Ozkose Z, Kavutçu M, Cavunt Bayraktar A, Bedirli A (2018). Sevoflurane exerts brain-protective effects against sepsis-associated encephalopathy and memory impairment through caspase 3/9 and Bax/Bcl signaling pathway in a rat model of sepsis. J. Int. Med. Res..

[70] Lin Z, Jin J, Shan X (2019). Fish oils protects against cecal ligation and puncture-induced septic acute kidney injury via the regulation of inflammation, oxidative stress and apoptosis. Int. J. Mol. Med..

[71] Akhurst RJ (2017). Targeting TGF-β signaling for therapeutic gain. Cold Spring Harb. Perspect. Biol..

[72] Pekgöz S, Asci H, Erzurumlu Y, Savran M, Ilhan I, Hasseyid N, Ciris M (2022). Nebivolol alleviates liver damage caused by methotrexate via AKT1/Hif1α/eNOS signaling. Drug Chem. Toxicol..

[73] Colak S, Gurlek B, Topcu A, Tumkaya L, Mercantepe T, Yilmaz A (2020). Protective effects of nebivolol on ovarian ischemia-reperfusion injury in rat. J. Obstet Gynaecol. Res..

[74] Gul R, Okla M, Mahmood A, Nawaz S, Fallata A, Bazighifan A, Alfayez M, Alfadda AA (2023). Comparison of the protective effects of nebivolol and metoprolol against LPS-induced injury in H9c2 cardiomyoblasts. Curr. Issues Mol. Biol..

[75] Gandhi C, Zalawadia R, Balaraman R (2008). Nebivolol reduces experimentally induced warm renal ischemia reperfusion injury in rats. Renal Fail..

