# Supplementary material for: Nebivolol ameliorates sepsis-evoked kidney dysfunction by targeting oxidative stress and TGF-β/Smad/p53 pathway
Source: Sci Rep. 2024 Jun 26;14:14735. doi: 10.1038/s41598-024-64577-5 (PMC11208533; doi:10.1038/s41598-024-64577-5)

TGF-β1 (25 KDa)

Repeat 1

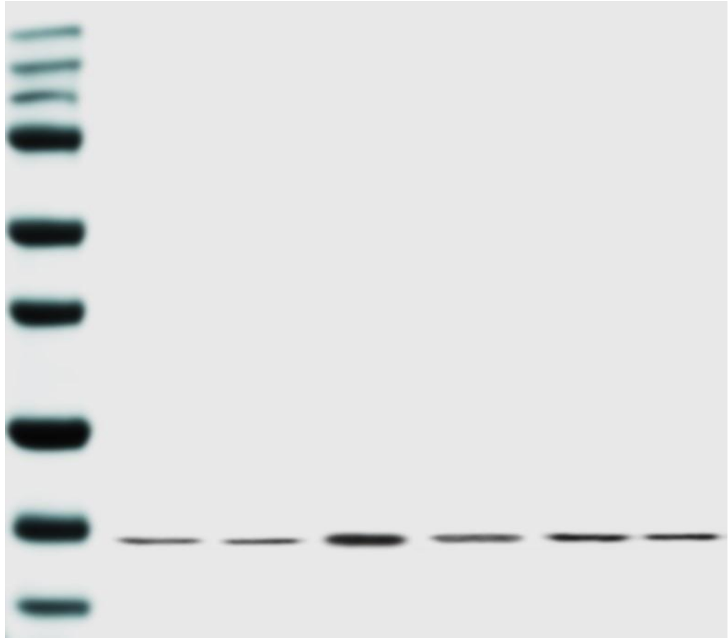

Repeat 2

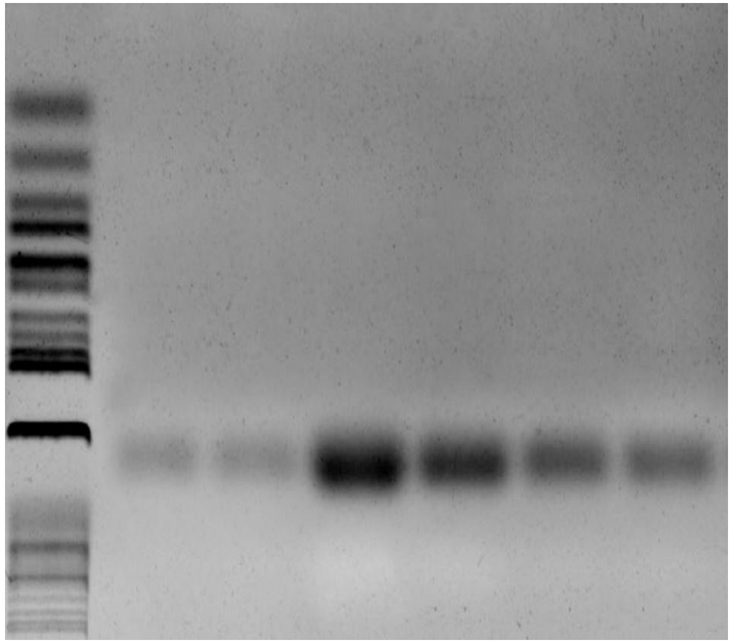

Repeat 3

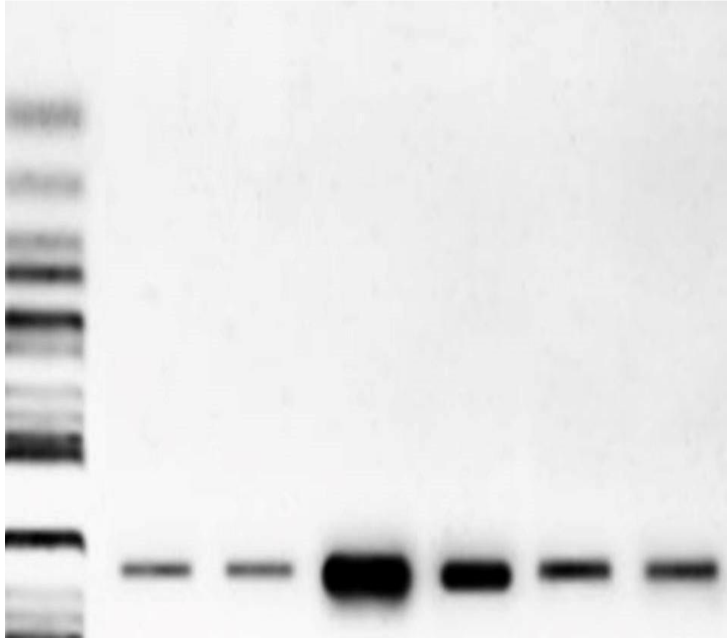

Smad 2/3 (52-60 KDa)

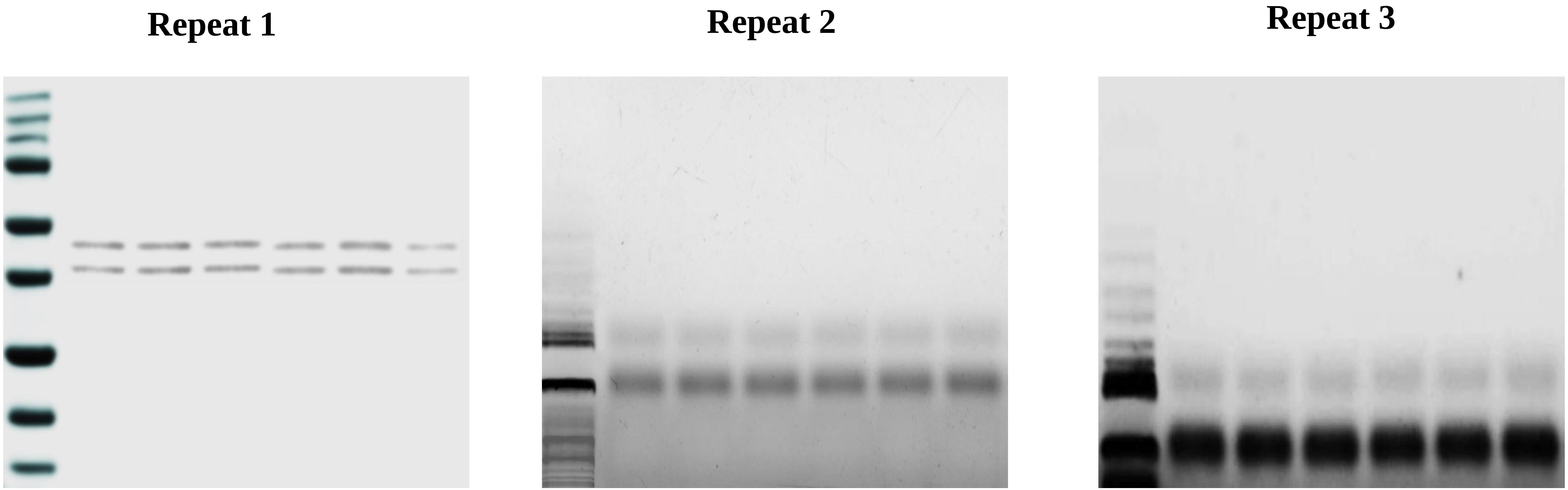

P-Smad 2/3 (52-60 KDa)

Repeat 1

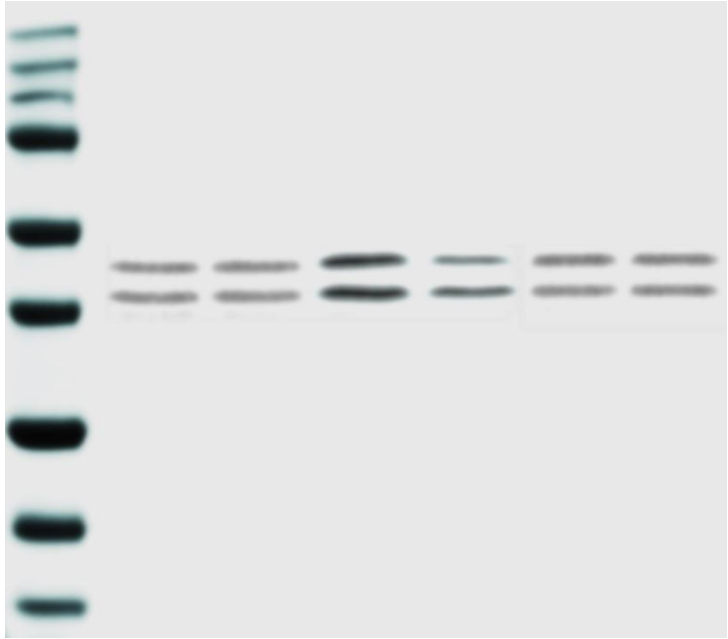

Repeat 2

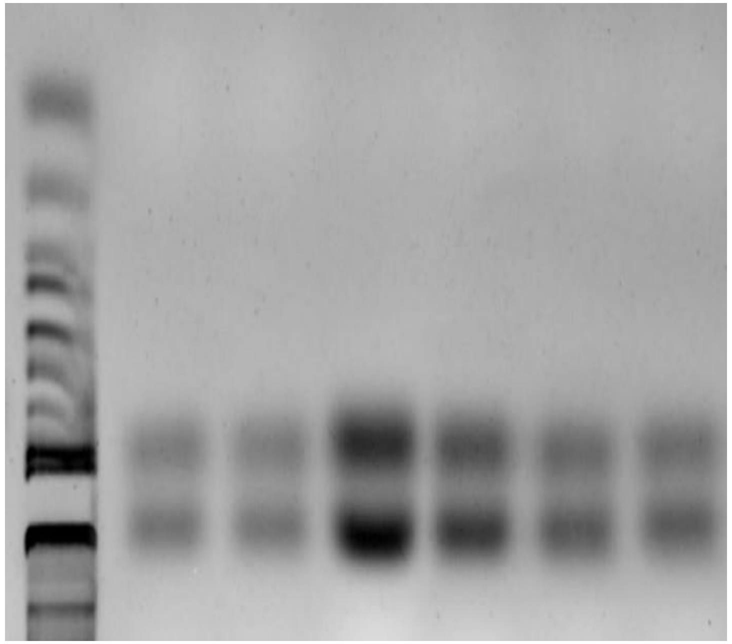

Repeat 3

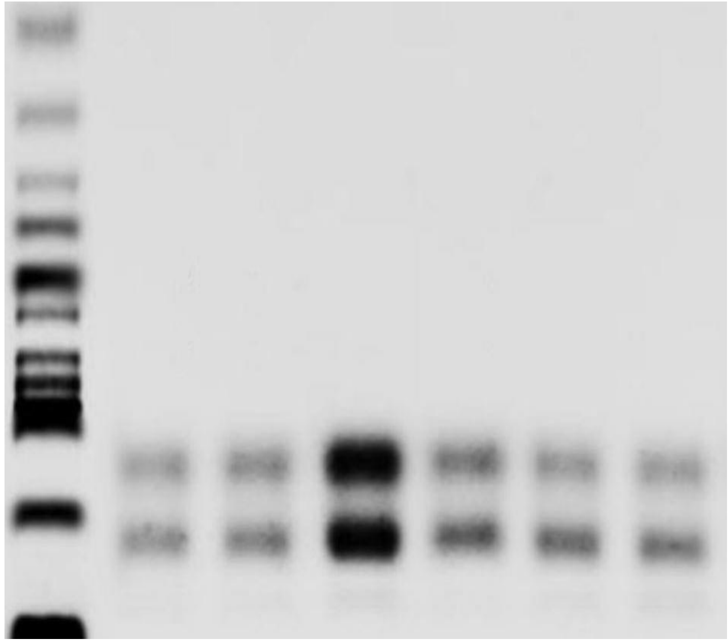

alpha-SMA (42 KDa)

Repeat 1

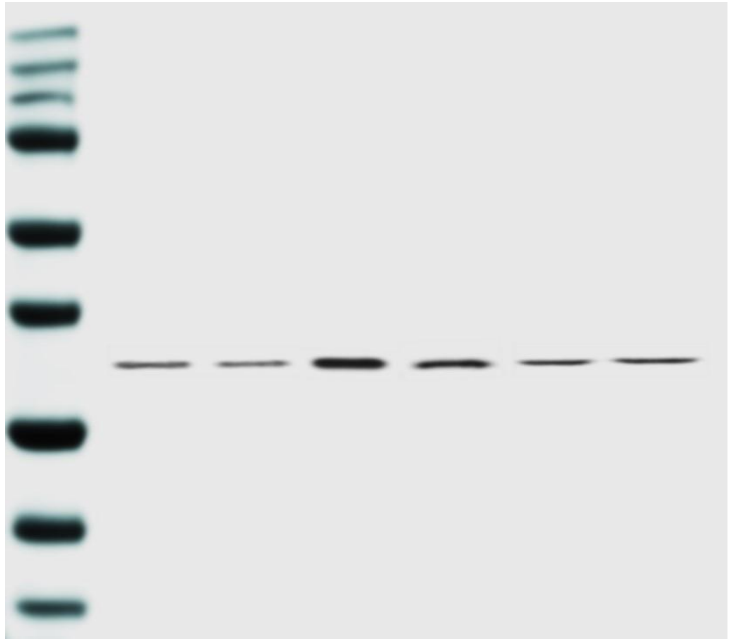

Repeat 2

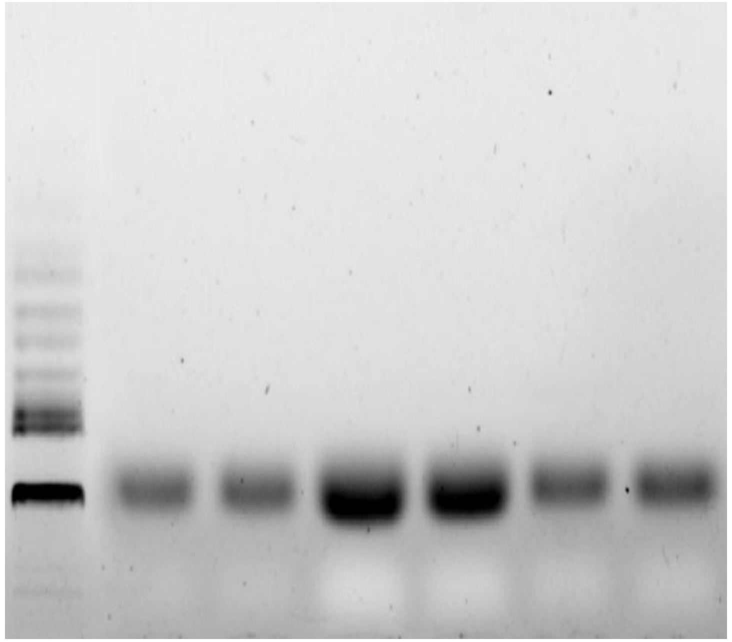

Repeat 3

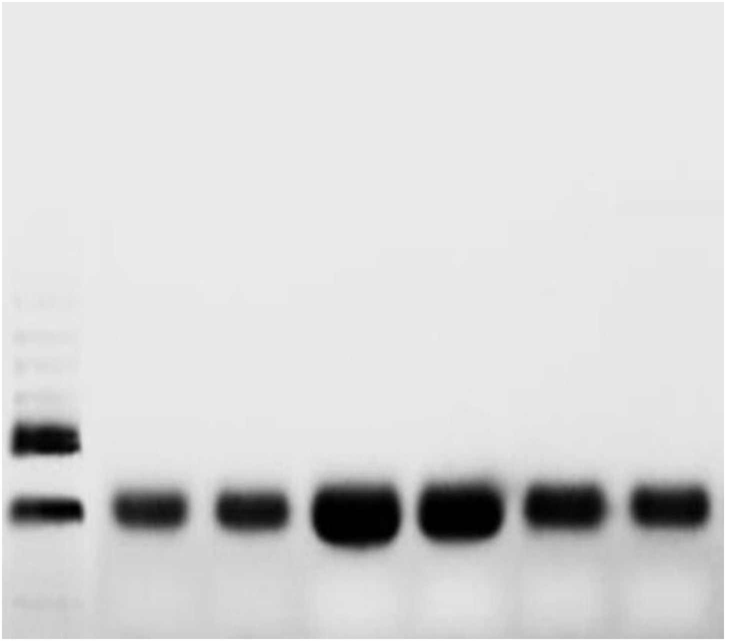

β-actin (43 KDa)

Repeat 1

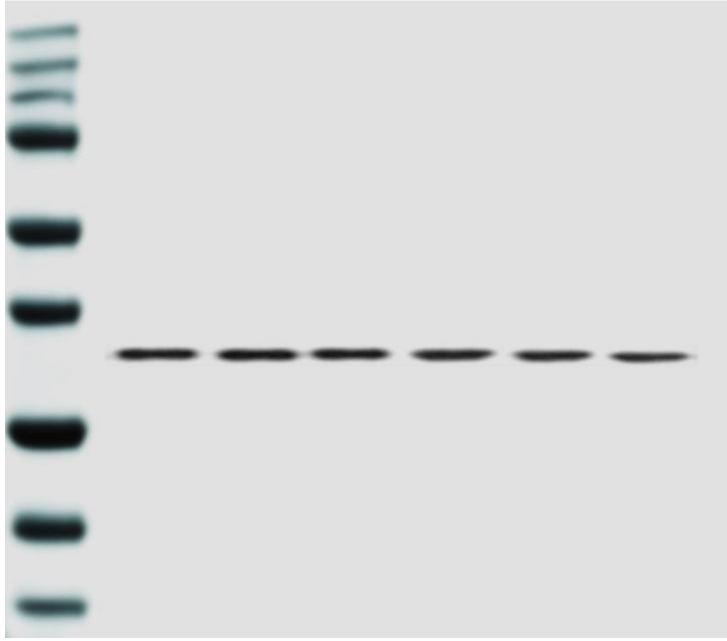

Repeat 2

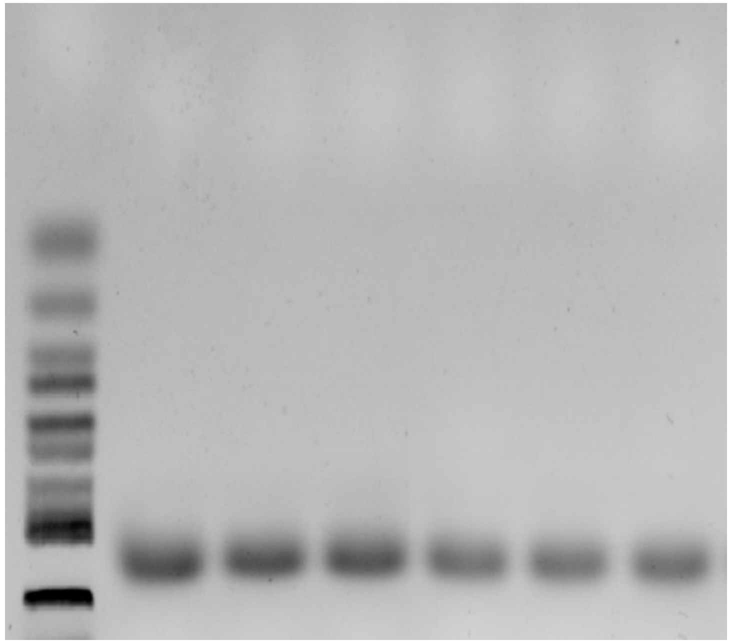

Repeat 3

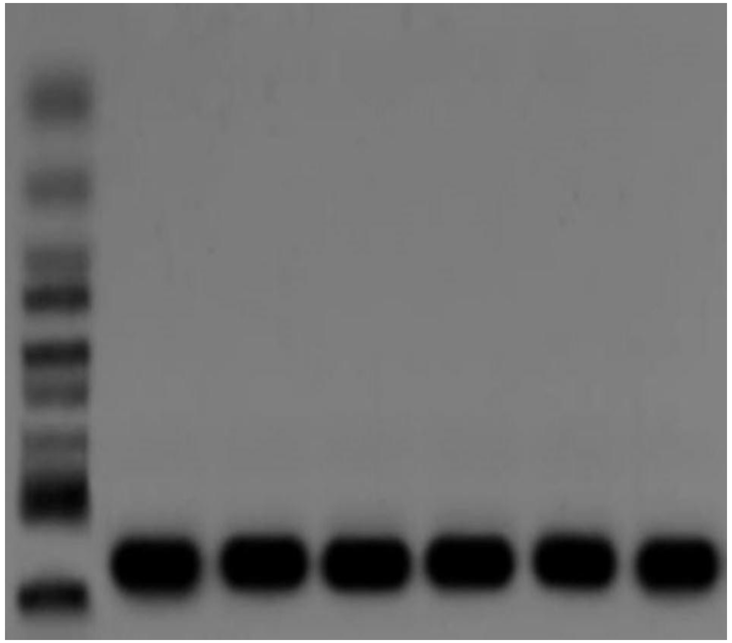

Supplement: Supplementary file 1 — Supplementary Information. [file 41598_2024_64577_MOESM1_ESM.pdf]
